# Role of Long Noncoding RNAs and Circular RNAs in Nerve Regeneration

**DOI:** 10.3389/fnmol.2019.00165

**Published:** 2019-06-28

**Authors:** Chun Yao, Bin Yu

**Affiliations:** Key Laboratory of Neuroregeneration of Jiangsu and Ministry of Education, Co-innovation Center of Neuroregeneration, Nantong University, Nantong, China

**Keywords:** long noncoding RNA, circular RNA, peripheral nerve injury, spinal cord injury, nerve regeneration

## Abstract

Nerve injuries may cause severe disability and affect the quality of life. It is of great importance to get a full understanding of the biological processes and molecular mechanisms underlying nerve injuries to find and target specific molecules for nerve regeneration. Numerous studies have shown that noncoding RNAs (ncRNAs) participate in diverse biological processes and diseases. Long noncoding RNAs (lncRNAs) and circular RNAs (circRNAs) are two major groups of ncRNAs, which attract growing attention. The altered expression patterns of lncRNAs and circRNAs following nerve injury suggest that these ncRNAs might be associated with nerve regeneration. This review will give a brief introduction of lncRNAs and circRNAs. We then summarize the current studies on lncRNAs and circRNAs following peripheral nerve injury and spinal cord injury (SCI). Typical lncRNAs and circRNAs are introduced to illustrate the diverse molecular mechanisms for nerve regeneration. In addition, we also discuss some issues to be addressed in future investigations on lncRNAs and circRNAs.

## Introduction

Injuries to the central nervous system (CNS) or peripheral nervous system (PNS) are a common clinical problem and cause inconvenience in daily life. Although PNS has an intrinsic regenerative ability following nerve injury compared with CNS, the axon regeneration speed is slow and the functional recovery is poor. To explore the complex biological processes underlying nerve injury and identify specific molecules in nerve regeneration will provide new therapeutic targets for nerve injury (Chan et al., 2014).

More than three-quarters of the human genome is transcribed with only less than 2% is coding for proteins, suggesting a potential RNA-based regulation network (Djebali et al., 2012; Fatica and Bozzoni, 2014). These RNAs that do not encode a protein, but have other cellular functions were termed as noncoding RNAs (ncRNAs; Cech and Steitz, 2014). In addition to ribosomal RNAs (rRNAs) and transfer RNAs (tRNAs), new classes of ncRNAs have been identified over the last two decades, including microRNAs (miRNAs), long noncoding RNAs (lncRNAs) and circular RNAs (circRNAs; Cech and Steitz, 2014; Qu et al., 2015).

NcRNAs could guide DNA synthesis, genome rearrangement and regulate gene expression during transcription, RNA processing and translation, emerging as a major source of biomarkers and targets for therapeutics in numerous diseases (Cech and Steitz, 2014; St Laurent et al., 2015). Previously, the role of miRNAs in nerve regeneration has been summarized (Yu et al., 2015). Recently, the dynamic alterations and functions of lncRNAs and circRNAs after nerve injury were also uncovered. LncRNA Kcna2 AS is increased in dorsal root ganglion (DRG) after peripheral nerve injury. Blocking Kcna2 AS expression could attenuate the development and maintenance of neuropathic pain by increasing Kcna2 (Zhao et al., 2013). Rat sciatic chronic constriction injury (CCI) induced a comprehensive expression profile of circRNAs, suggesting a potential role of circRNAs in neuropathic pain (Cao et al., 2017). In addition to neuropathic pain (Li et al., 2019; Wu et al., 2019), lncRNAs and circRNAs could also regulate nerve regeneration after nerve injury.

**Figure 1:**
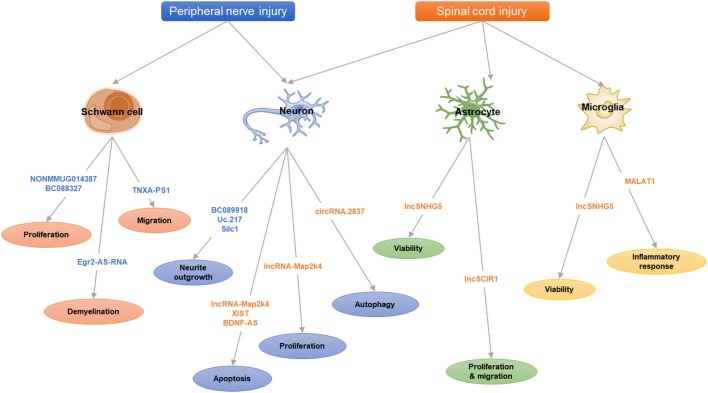
The scheme of long noncoding RNAs (lncRNAs) and circular RNAs (circRNAs) in nerve injury.

Here, we review the recent studies on lncRNAs and circRNAs following nerve injuries (peripheral nerve injury and spinal cord injury, SCI; Table 1) and summarize some key lncRNAs and circRNAs (Table 2 and Figure 1) to illustrate their functional mechanisms in nerve regeneration.

**Table 1:** List of studies on long noncoding RNA (lncRNA) and circular RNA (circRNA) alteration after nerve injury.

Injury model	Tissue	Time points post injury	Methods	Reference
**LncRNA**				
**•Peripheral nerve injury**				
Rat sciatic nerve transection	L4-L6 DRGs	0, 1, 4, 7 days	Microarray	Yu et al. (2013)
Mouse sciatic nerve crush	DRGs	0, 1, 4, 7 days	RNA-Seq	Perry et al. (2018)
Mouse sciatic nerve crush	Distal sciatic nerve	0, 3, 7, 14 days	Microarray	Pan et al. (2017b)
Mouse sciatic nerve transection itransection	Distal sciatic nerve	uncut, 7 days	RNA-Seq	Arthur-Farraj et al. (2017)
IL-22 treated rat SCs	SCs		RNA-Seq	Xu et al. (2017)
Rat sciatic nerve transection	Sciatic nerve	8 weeks	Microarray	Wang et al. (2018)
Rat sciatic nerve crush	Sciatic nerve	0, 1, 4, 7 days	Microarray	Yao et al. (2018)
**•Spinal cord injury**				
Rat contusion SCI	Epicenter spinal cord	0, 1, 4 and 7 days	RNA-Seq	Wang J. et al. (2015)
Mouse contusion SCI	Epicenter spinal cord	0, 1, 3 days, 1 and 3 weeks	Microarray	Ding et al. (2016)
Rat contusion SCI	Epicenter spinal cord	0, 1, 3, 6 months	RNA-Seq	Duran et al. (2017)
Rat contusion SCI	Epicenter spinal cord	2 days	Microarray	Shi et al. (2019)
**CircRNA**				
Rat traumatic SCI	Spinal cord	3 days	Microarray	Qin et al. (2018)
Rat traumatic SCI	Spinal cord	6 h	RNA-Seq	Zhou et al. (2019)
Mice sciatic nerve injury	Gastrocnemius muscles	0, 1, 2, 4, 8 weeks	RNA-Seq	Weng et al. (2018)
Rat sciatic nerve compression	Sciatic nerve	1 day	RNA-Seq	Zhou Z.-B. et al. (2018)

**Table 2:** Summary of the functions and mechanisms of lncRNAs and circRNAs in nerve injury.

LncRNA	Injury model	Expression	Function	Mechanism	Reference
**•Peripheral nerve injury**					
BC089918	Rat sciatic nerve transection	down	Negatively regulate neurite outgrowth	Unclear	Yu et al. (2013)
Uc.217	Rat sciatic nerve transection	down	Negatively regulate neurite outgrowth	Affect the expressions of genes involved in nerve regeneration	Yao et al. (2015)
Silc1	Mouse sciatic nerve crush	up	Depletion of Silc1 impair neurite outgrowth	Activate in *cis* the expression of transcription factor Sox11	Perry et al. (2018)
NONMMUG014387	Rat sciatic nerve transection	up	Promote SC proliferation	Increase Cthrc1 expression and activate Wnt/PCP pathway	Pan et al. (2017a)
BC088327	Rat sciatic nerve transection	up	Promote SC proliferation	Synergic with heregulin-1β	Wang et al. (2018)
TNXA-PS1	Rat sciatic nerve crush	down	Downregulation of TNXA-PS1 promote SC migration	Act as a ceRNA to affect Dusp1 expression	Yao et al. (2018)
Egr2-AS-RNA	Mouse sciatic nerve transection	up	Induce demyelination	Inhibit Egr2 expression	Martinez-Moreno et al. (2017)
**•Spinal cord injury**					
lncSCIR1	Rat contusion SCI	down	Negatively regulate astrocyte proliferation and migration.	Affect the expressions of Adm, Bmp7, Snca and Wnt3	Wang J. et al. (2015)
lncSNHG5	Rat contusion SCI	up	Enhance astrocytes and microglia viability	Interact with KLF4	Jiang and Zhang (2018)
lncRNA-Map2k4	Mouse contusion SCI	down	Promote neuron proliferation and inhibit apoptosis	Through an miR-199a/FGF1 pathway	Lv (2017)
XIST	Rat contusion SCI	up	Induce neuronal apoptosis	Negatively modulate PI3K/AKT pathway by decreasing miR-494 and increasing PTEN expression	Gu et al. (2017)
BDNF-AS	Rat ASCI and hypoxia cellular model	up	Promote neuronal cell apoptosis	Sponge miR-130b-5p to regulate PRDM5	Zhang et al. (2018a)
DGCR5	Rat ASCI and hypoxia cellular model	down	Suppress neuronal apoptosis	Bind and negatively regulate PRDM5	Zhang et al. (2018b)
MALAT1	Rat ASCI	up	Regulate inflammatory response of microglia	*Via* miR-199b/IKKβ/NF-κB pathway	Zhou H.-J. et al. (2018)
**CircRNA**
circRNA 2837	Rat sciatic nerve compression	down	Regulate autophagy in neurons	Serve as a miRNA sponge for the miR-34 family	Zhou Z.-B. et al. (2018)

## A Brief Glance at LncRNAs and CircRNAs

### Origins, Characteristics and Classifications

LncRNAs are a class of ncRNAs longer than 200 nt, usually capped, polyadenylated and spliced without significant protein-coding capacity (Rinn and Chang, 2012). The origins of lncRNAs are diverse. Besides metamorphosis from pre-existing protein-coding sequence, lncRNAs can also emerge from chromosome rearrangement, retrotransposition, tandem duplication and transposable element sequences insertion (Ponting et al., 2009). LncRNAs have been found to be transcribed from various genome regions, including promoter upstream regions, enhancers, intergenic regions and the opposite strand of protein-coding genes. Some lncRNA species are generated by unique biogenesis pathways, such as RNase P cleavage and capping by small nucleolar RNA (snoRNA)-protein (snoRNP) complexes at their ends (Wu et al., 2017). Previously, based on the genomic location relative to neighboring protein-coding genes, lncRNAs are classified into sense, antisense, bidirectional, intronic and intergenic lncRNAs (Peng et al., 2018). In addition, there are new lncRNA species according to the association with other DNA elements or based on unique structures, including the promoter up-stream transcripts (PROMPTs), enhancer-associated RNAs (eRNAs), sno-lncRNAs and so on (St Laurent et al., 2015).

Circular RNAs (circRNAs) are an increasingly appreciated class of ncRNA. Unlike linear RNAs, circRNAs are characterized by a covalently closed continuous loop without 5′-3′ polarity or a polyadenylated tail (Qu et al., 2015). Compared to the diverse origins of lncRNAs, circRNAs usually originate from protein-coding genes and complete exons (Pamudurti et al., 2017). Eukaryotic circRNAs are mainly produced during splicing, catalyzed by either the spliceosomal machinery or by groups I and II ribozymes (Vicens and Westhof, 2014). In addition to circRNAs formed from exon back-splicing circularization, there are various types of circRNAs existed according to different biogenesis mechanisms, such as circular viral RNA genomes, circRNA intermediates, spliced introns and exons production (Qu et al., 2015; Chen, 2016). Due to the lack of free ends, circRNAs are resistant toward exonucleases (Vicens and Westhof, 2014). Besides that property, circRNAs also have potentials for rolling circle amplification, rearranging genomic sequences and constraining RNA folding (Vicens and Westhof, 2014). As the first known function of a circRNA is miRNA sponge, circRNAs are predicted as ncRNAs regulating miRNAs. However, some of the circRNAs are translated into polypeptides (Legnini et al., 2017; Pamudurti et al., 2017).

### Functions and Mechanisms

Numerous studies have revealed the role of lncRNAs and circRNAs in disease pathological progress, diagnosis, and prognosis (Wan et al., 2017; Han et al., 2018). In the nervous system, lncRNAs could take part in brain development, neuron maintenance and differentiation, as well as synaptic plasticity, cognitive function and memory (Wu et al., 2013). In addition, dysregulated lncRNAs were also found in neurodegenerative diseases in human, such as lncRNA BACE1-AS and BC200 (Alzheimer’s disease, AD), PINK1-AS (Parkinson’s disease), NEAT1 and MEG3 (Huntington’s disease), AS C9ORF72 (amyotrophic lateral sclerosis) and so on (Wan et al., 2017).

CircRNAs are enriched in mammalian brain, conserved and dynamically expressed during neuronal differentiation (Rybak-Wolf et al., 2015). Expressions of circRNAs in mouse hippocampus and prefrontal cortex, regions involved in memory formation and learning, were illustrated (Chen B.-J. et al., 2018). In adult rat subventricular zone (SVZ), one SVZ-specific circRNA (rat_circ:chr15:9915223-9915671) was identified to bind miR-138-5p as a potential negative regulator in neural stem cell proliferation (Xie et al., 2018). CircRNA CDR1as (ciRS-7) was exclusively highly expressed in brain tissues and served as a potential circular miR-7 sponge in neuronal tissues (Memczak et al., 2013). In human AD brain tissues, ciRS-7 was decreased with increased expression of miR-7 and could downregulate expression of several AD-relevant genes, such as the ubiquitin conjugase (UBE2A) protein (Zhao et al., 2016).

The functional mechanisms of lncRNAs and circRNAs are complicated. Briefly, lncRNAs localized in nucleus could interact with chromatin complexes, modulate proteins and enzyme cofactors, bind DNA/RNA-binding proteins or interact directly with DNA to form R-loops and triple helixes, working as key regulators for transcriptional programs (Marchese et al., 2017). On the other hand, cytoplasmic lncRNAs could also interact with proteins to form complex, regulating a set of cytoplasmic events (e.g., protein localization and turnover, mRNA translation and stability). Cytoplasmic lncRNAs can also act as sponges of miRNAs (competing endogenous RNA, ceRNA) and alleviate the negative effects of miRNAs on its target genes (Noh et al., 2018).

Comparing with lncRNAs, the large majority of circRNAs are in the cytoplasm. These circRNAs can function as miRNA or lncRNA sponges to indirectly regulate mRNA expression through a complex RNA communication network (Wang Y.-H. et al., 2015; Chen, 2016; Adams et al., 2017). Gene regulatory axis of lncRNAs-miRNAs-circRNAs has been described in the brain recently (Kleaveland et al., 2018). It reported that lncRNA Cyrano could bind with miR-7 to trigger miR-7 degradation, preventing repression of miR-7 targeted mRNAs and promoting circRNA Cdr1as accumulation. On the other hand, miR-7 could lead to destruction of Cdr1as with another miRNA miR-671. These ncRNAs collaborated to establish a sophisticated regulatory network. In addition, some exon-intron circRNAs could also interact with transcription complexes to affect gene transcription (Barrett and Salzman, 2016).

## LncRNAs in Nerve Injury

### Peripheral Nerve Injury

Injuries to peripheral nerves by trauma or acute compression may lead to axon continuity interruption, nerve degeneration and neuron death, resulting in partial or total loss of nerve functions (Navarro et al., 2007). Unlike CNS, the PNS has a regenerate ability after injury due to the intrinsic growth capacity of neurons and a permissive microenvironment mainly provided by Schwann cells (SCs; Barton et al., 2017). The sciatic nerve injury model is a classic model for investigating effects of peripheral nerve regeneration. After sciatic nerve injury, in the distal segment of the lesion, a progressive degeneration (Wallerian degeneration) is occurring, breaking down both axons and myelin. This process is accompanied by the dedifferentiation of SCs and activation of immune response (Chen et al., 2015). The dedifferentiated SCs could replenish the damaged tissues by proliferation, clear myelin debris, secrete neurotrophic factors and form Bungner band, providing a favorable environment for axon regeneration and ensuing remyelination (Jessen and Mirsky, 2016). In the proximal nerve stump, the damaged neurons shift their states from neurotransmission to regeneration, which is triggered by a sequence of molecular responses, including calcium influx, activation of protein kinase pathways and gene expression by transcription factors (Wu and Murashov, 2013). Nerve regeneration following peripheral nerve injury is a complicated process, accompanied with multiple signaling pathways and genes, such as Neuregulin 1 signaling, Gpr126 signaling, Erk signaling, PI3K-GSK3 signaling, DLK-JNK signaling, Wnt signaling and so on (Glenn and Talbot, 2013; Zhang and Zhou, 2013).

#### Intrinsic Regeneration Ability of Neurons

The survival and transition into a regenerative state of injured neurons are critical for nerve regeneration. DRG neurons provide a favorable model for mammalian axon regeneration investigation among PNS neurons (Zhang and Zhou, 2013). Yu et al. (2013) reported the altered lncRNA expressions in DRGs after rat sciatic nerve injury. By lncRNA microarray, they identified 105 lncRNAs dysregulated in L4-L6 DRGs at 0, 1, 4 and 7 days post sciatic nerve transection. In a mouse sciatic nerve crush model, Perry et al. (2018) identified dysregulated lncRNAs expressed during neuroregeneration in DRGs by RNA-Seq.

From these lncRNA profiles after peripheral nerve injury, BC089918 was identified to have a negative effect on neurite outgrowth *in vitro*. However, its regulatory mechanism is unclear (Yu et al., 2013). Another lncRNA, uc.217, was also selected for validation. The data showed that uc.217 was down-regulated in DRG neurons after sciatic nerve injury and could regulate neurite outgrowth in cultured DRG neurons by affecting the expressions of regeneration-associated genes, Gal and Vip. Besides that, uc.217 could serve as a sponge RNA to regulate Sema3d and Smad7 expressions, which are genes involved in nerve regeneration (Yao et al., 2015). Experiments suggested that lncRNAs, Silc1 and Norris1, could affect neurite outgrowth *in vitro*. In addition, mice with Silc1 deletion had a delayed regeneration after sciatic nerve crush. Further investigations showed that Silc1 could affect neuroregeneration by activating in *cis* the expression of Sox11, which is a well-known transcription factor that could modulate peripheral nerve regeneration (Jankowski et al., 2009; Perry et al., 2018).

#### Schwann Cell Phenotype Modulation

As the major glial cells in PNS, SCs also play an important role in peripheral nerve regeneration. Upon injury, SCs will dedifferentiate, proliferate and migrate to form bands of Bungner, providing guidance cues for regeneration. In addition, SCs will also clear myelin debris and secrete neurotrophic factors to facilitate nerve injury repair (Glenn and Talbot, 2013). Previously, numerous studies have confirmed the role of miRNAs in SC modulation, such as miR-221-3p, miR-182, miR-9 and miR-132 (Yao et al., 2016; Zhao L. et al., 2018). Similar to miRNAs, lncRNAs also take part in regulating SCs. Profiles of lncRNA expression alterations in sciatic nerve after injury or in SCs were identified in different conditions (Table 1; Arthur-Farraj et al., 2017; Pan et al., 2017b; Xu et al., 2017; Yao et al., 2018; Wang et al., 2018).

Further investigation identified several potential lncRNAs for SC modulation. LncRNA NONMMUG014387 was upregulated after peripheral nerve injury and could promote SC proliferation by increasing collagen triple helix repeat containing 1 (Cthrc1) and activating Wnt/PCP pathway (Pan et al., 2017a). LncRNA BC088327 was increased with the exposure time of heregulin-1β in SCs under hypoxic conditions. Knockdown of BC088327 expression could significantly suppress cell viability in the presence of heregulin-1β. This suggested that BC088327 could play a synergistic role with heregulin1β in SC proliferation during peripheral nerve repair (Wang et al., 2018). Recently, our group identified that lncRNA TNXA-PS1 (NR_024118) could affect SC migration by acting as a sponge RNA for miR-24-3p/miR-152-3p. After sciatic nerve injury, TNXA-PS1 was down-regulated, releasing miR-24-3p and miR-152-3p previously bound. These miRNAs could interact with the 3′-UTR of Dual specificity phosphatase 1 (Dusp1) and reduce Dusp1 expression, promoting SC migration (Yao et al., 2018).

Besides modulating SC proliferation and migration, lncRNAs could also take part in SC myelination. Previous studies have shown the crucial role of transcription factor Egr2 in the control of SC myelination (Ghislain et al., 2002; Le et al., 2005). The expression of lncRNA Egr2-AS-RNA, an anti-sense RNA to the promoter of Egr2, was increased after peripheral nerve injury. This will lead to the decline of Egr2 expression and induce demyelination by recruiting H3K27ME3, AGO1, AGO2 and EZH2 on Egr2 promoter (Martinez-Moreno et al., 2017). Upstream exploration indicated that Egr2-AS-RNA expression is regulated by ERK1/2 signaling. Nerve injury inhibits the NRG1-ERK1/2 signaling axis, which dephosphorizes YY1 and elevating Egr2-AS-RNA expression.

### Spinal Cord Injury

Comparing to the PNS, the CNS has limited regeneration ability. It was estimated that about 3 million people suffered from traumatic SCI, with about 180,000 new cases annually in the worldwide (Lee et al., 2014). SCI is much more complicated due to extensive cell loss, axonal disruption, glial scar and a shortage of growth-permissive factors (Estrada and Müller, 2014). After a primary mechanical injury (damage to neurons, axons, and glia at injury sites), a subsequent secondary injury will develop within hours or days, including ischemia, edema, inflammation and vascular changes (Silva et al., 2014). During this phage, inflammatory cells, such as macrophages, microglia, T-cells and neutrophils, will infiltrate the injury site and release inflammatory cytokines. The architectural disruption and glial scarring formed by uncontrolled reactive astrogliosis impair directed axonal regrowth (Ahuja and Fehlings, 2016). Extensive temporal gene expression changes were occurred during these processes. In addition, the role and implication of miRNAs in SCI have been widely investigated (Bhalala et al., 2013; Ning et al., 2014). Recent years, the alterations of lncRNAs after SCI at different time points were extensively identified (Wang J. et al., 2015; Ding et al., 2016; Duran et al., 2017; Shi et al., 2019), suggesting that lncRNAs might also take part in SCI.

#### Reactive Astrocytes

One of the main barriers to SCI regeneration is glial scar, which is composed primarily of astrocytes (Silver and Miller, 2004). In response to nerve injury, the astrocytes are hypertrophic reactivated. The enlarged and entangled reactive astrocytes will develop then into a rubbery, tenacious barrier for nerve regeneration. On the other hand, astrocyte gliosis might also provide permissive functions for CNS injury, such as neuroprotection, facilitating blood brain barrier repair, limiting the spread of inflammatory cells and so on (Sofroniew and Vinters, 2010).

LncSCIR1 was down-regulated post SCI and knockdown of lncSCIR1 could promote astrocyte proliferation and migration (Wang J. et al., 2015). However, the regulatory mechanism of lncSCIR1 was unclear. LncRNA SNHG5 (small nucleolar RNA host gene5) has been widely studied in tumorigenesis (Damas et al., 2016; Zhao et al., 2017). It can regulate gene expression by sponging miRNAs (He et al., 2017) or stabilizing target transcripts (Damas et al., 2016). Recently, lncSNHG5 was also identified to enhance the viability of astrocytes and microglia after SCI through SNHG5/KLF4/eNOS axis (Jiang and Zhang, 2018). Kruppel-like factor 4 (KLF4), an inflammation associated molecule induced in reactive astrocytes and microglia, might be positively regulated by SNHG5 as a sponge RNA for miR-32 (Zhao et al., 2017).

#### Neuron Survival

Neuronal survival and axon regeneration are two key decisions of injured neurons (He and Jin, 2016). A number of neurotrophins (NGF, BDNF and NT-3) and miRNAs (miR-486, miR-20a, miR-21) have been identified to regulate neuronal survival after SCI (Bhalala et al., 2013; Ning et al., 2014; Keefe et al., 2017).

Fibroblast growth factor 1 (FGF1) is a powerful neurotrophic factor for nerve regeneration (Tsai et al., 2015). LncRNA-Map2k4 (ENSMUST00000138093) was declined after SCI and could affect neuron proliferation and apoptosis through an miR-199a/FGF1 pathway. Knockdown of LncRNA-Map2k4 will inhibit FGF1 expression by up-regulating miR-199a (Lv, 2017). Some classical lncRNAs involved in cancer progress and neurological diseases can also affect neuronal survival after SCI. By retrieving the microarray data in the GEO dataset, lncRNA XIST was found significantly increased after SCI. Down-regulating lncRNA XIST could improve locomotor activity and attenuate neuronal apoptosis after SCI by activating PI3K/AKT pathway through mopping up of miR-494 (Gu et al., 2017). After acute spinal cord injury (ASCI), lncRNA BDNF-AS were up-regulated. BDNF-AS will then sponge miR-130b-5p and increase PRDM5 expression, leading to neuronal apoptosis (Zhang et al., 2018a). LncRNA DGCR5 is a significant neural lncRNA involved in a number of neurological disorders, such as Huntington’s disease (Johnson, 2012). Recent studies found that DGCR5 was also down-regulated in ASCI model and in neurons treated with hypoxia. DGCR5 could inhibit neuronal apoptosis by directly binding and negatively regulating PRDM5 expression. Overexpression of DRCR5 could ameliorate ASCI in rats with elevated BBB score (Zhang et al., 2018b).

#### Inflammation

After SCI, inflammation is triggered with activated microglia, hematogenous macrophages and other inflammatory cells recruited to the lesion site, leading to secondary injury (Assinck et al., 2017). The activated immune cells will secret proinflammatory factors and contribute to cell death by producing ROS (Tran et al., 2018). On the other hand, some macrophages can promote nerve regeneration by secretion of anti-inflammatory factors. Besides lncSNHG5, which we have discussed above, lncRNA MALAT1 also take part in microglia activation. After acute SCI, MALAT1 expression was significantly increased, resulting in the activation of IKKβ/NF-κB signaling pathway. MALAT1 could also promote the secretion of proinflammatory cytokines TNF-α and IL-1β in microglia through down-regulating miR-199b (Zhou H.-J. et al., 2018).

## CircRNAs in Nerve Injury

Recently, the investigation of circRNAs in nerve injury is increasing. A number of studies have revealed circRNA expression patterns in the models of traumatic brain injury and neuropathic pain by microarray and RNA-seq (Zhou et al., 2017; Chen Z. et al., 2018; Zhao R.-T. et al., 2018). One-hundred and eighty-eight differentially expressed circRNAs (68 up-regulated and 120 down-regulated) were identified in rat spinal cord at 14 days after spared nerve injury. Among them, circ_0006928 was found might regulate neuron apoptosis by binding miR-184 (Zhou et al., 2017). circRNAs-Filipi1l, which was negatively regulated by miRNA-1224 *via* binding and splicing in the Ago2-dependent manner, was increased in chronic inflammation pain and could regulate nociception by targeting Ubr5 (Pan et al., 2019).

However, the roles of circRNAs in nerve regeneration remain unknown. The expression profiles of circRNAs in rat spinal cord after traumatic SCI have been identified (Qin et al., 2018; Zhou et al., 2019). As for peripheral nerve injury, there is one report for the whole transcriptome involved in denervated muscle atrophy after peripheral nerve injury (Weng et al., 2018). In addition, differentially expressed circRNAs in a rat sciatic nerve compression model were identified and one down-regulated circRNA, circRNA.2837, was found to regulate autophagy in neurons by serving as a miRNA sponge for the miR-34 family (Zhou Z.-B. et al., 2018). Silencing circRNAs.2837 could induce autophagy in primary spinal neurons by targeting miR-34a.

## Conclusion and Perspective

This review aims to summarize the recent progress of lncRNAs and circRNAs in nerve regeneration. From this review article, we could find that at present, most of the studies on lncRNAs and circRNAs in nerve regeneration were focused on microarray and RNA-sequencing data analysis. These studies always first conduct an animal model of nerve injury and then dissect nerve tissue or cells for microarray or RNA-sequencing to obtain significantly differentially expressed lncRNAs and circRNAs. Further, bioinformatic analyses will be performed to obtain enriched biological processes and signaling pathways of these ncRNAs and their regulated genes. In addition, one or two dysregulated ncRNAs will be selected for further qRT-PCR expression validation and lncRNAs/circRNAs-miRNA-gene regulated networks will be constructed to find target genes of certain lncRNA/circRNA.

Though a set of dysregulated lncRNAs after peripheral nerve injury and SCI has been reported, a few lncRNAs among them received further investigation and the mechanisms underlying the regulation role of lncRNAs in nerve injury are still unclear. A few number of studies got a deep investigation of certain lncRNA/circRNA to explore their characteristics, function and regulation mechanisms. Serving as a sponge RNA or binding proteins directly are two major mechanisms involved in the regulation role of lncRNAs in nerve regeneration till now.

In future investigation, there are some issues to be addressed: (1) The detailed mechanisms underlying the role of lncRNAs/circRNAs. We need to illustrate how lncRNAs/circRNAs regulate certain genes. Which segment of lncRNAs/circRNAs plays the critical regulatory role? (2) The interaction networks between lncRNAs-miRNAs-circRNAs and circRNAs-miRNAs-mRNAs. Recently, gene regulatory axis of lncRNAs-miRNAs-circRNAs has been described in the brain (Kleaveland et al., 2018). The possibility of interaction between circRNAs and lncRNAs after nerve injury deserves our further attention. In addition, more solid evidence should be provided to confirm the interaction between lncRNAs/circRNAs, miRNAs and target genes, such as Ago2 immunoprecipitation. (3) The upstream of lncRNAs/circRNAs. From what we have reviewed, the majority of these studies are focused on the function of lncRNAs/circRNAs after nerve injury. However, how nerve injuries trigger the expression alteration of lncRNAs/circRNAs? Which signaling or genes affect the dysregulation of lncRNAs/circRNAs? These upstream regulators of lncRNAs/circRNAs will be another interesting research topic. (4) The application of lncRNAs/circRNAs in nerve injury repair. Preclinical and clinical trials based on miRNA therapeutics have been conducted (Wahid et al., 2010). Some lncRNAs/circRNAs have been identified as prognostic markers in cancers (Tian and Xu, 2015; Lyu and Huang, 2017; Xiong et al., 2017). How could we apply lncRNAs/circRNAs to clinical nerve injury repair? Till now, the majority of studies on lncRNAs and circRNAs in nerve regeneration were based on animal models. Could these ncRNAs be used in clinical therapeutics? A recent study reported synthetically conserved lncRNAs in human, mouse, and rat by analyzing lncRNAs expressed in injured nerves of animal models and lncRNAs in human sensory neurons derived from iPSCs (Baskozos et al., 2019). In addition, circRNAs are also expected to be conserved between species (Rybak-Wolf et al., 2015). These studies showed the conservation of lncRNAs and circRNAs between species, which will facilitate the clinical transformation for lncRNAs and circRNAs in nerve injury. More efforts are needed to develop lncRNAs/circRNAs based clinical therapeutics for nerve injury.

## Author Contributions

CY conceived and wrote the review article. BY revised the article.

## Conflict of Interest Statement

The authors declare that the research was conducted in the absence of any commercial or financial relationships that could be construed as a potential conflict of interest.
